# A Maximum 34% Substitution of Fish Meal by Soybean Meal Is Recommended for *Rhynchocypris lagowskii* Dybowski Cultured in Biofloc Systems: Evidence From Growth and Intestinal Barrier Function

**DOI:** 10.1155/anu/5561983

**Published:** 2026-07-28

**Authors:** Zhi-Yong Yang, Deng-Lai Li, Ke-xin Wang, Li-Fang Wu, Rui Zhu, Qiong Wu

**Affiliations:** ^1^ College of Animal Science and Technology, Jilin Agricultural University, Changchun, China, jlau.edu.cn; ^2^ Testing Center of Quality and Safety in Aquatic Product, Changchun, China; ^3^ College of Life Sciences, Longyan University, Longyan 364012, China, lyun.edu.cn; ^4^ Tianjin Academy of Agriculture Sciences, Tianjin 300380, China

**Keywords:** antioxidant capacity, biofloc, growth performance, immune function, *Rhynchocypris lagowskii* Dybowski

## Abstract

In this study, glucose was used as the carbon source for the bioflocs in each group, with a carbon‐to‐nitrogen ratio (C/N) of 20 : 1. Juvenile *Rhynchocypris lagowskii* Dybowski (6.89 ± 0.02 g) were used as the experimental animals. Soybean meal (SBM) protein replaced 0.0% (CK), 17.0% (B17), 34.0% (B34), 51.0% (B51), and 68.0% (B68) of fish meal protein to formulate five isonitrogenous and isolipidic experimental diets for an 8‐week feeding trial. Under biofloc technology (BFT) conditions, the B68 group exhibited significantly lower growth performance (final weight [FW], weight gain rate [WGR], specific growth rate [SGR], feed efficiency ratio [FER], protein efficiency ratio [PER]) and muscle crude protein content compared to the CK group (*p* < 0.05). The B51 group showed significantly reduced activities of hepatopancreatic and intestinal protease (PRS), as well as of superoxide dismutase (SOD), catalase (CAT), glutathione peroxidase (GSH‐PX), and total antioxidant capacity (T‐AOC) in the distal intestine (DI). Meanwhile, the malondialdehyde (MDA) content in the DI was significantly increased in both the B51 and B68 groups (*p* < 0.05). The B68 group showed significantly increased serum GPT, GOT, and immunoglobulin M (IgM) levels, and significantly decreased hepatopancreatic GPT and GOT activities as well as serum complement 3 (C3), complement 4 (C4), and LSZ levels (*p* < 0.05). In the B68 group, the expression levels of multiple genes were significantly downregulated (*p* < 0.05): IGF‐1 in the mid intestine (MI) and DI, S6K in the hepatopancreas and DI, and ZO‐1, mTOR, IL‐10, TGF‐β, Nrf2, Maf, HO‐1, CAT, GSH‐PX, and occludin‐a in the DI. In contrast, significant up‐regulation was observed for TNF‐α (in MI and DI), as well as for 4E‐BP, NF‐κB, IL‐1β, IL‐8, and Keap1 (*p* < 0.05). In the BFT, SBM substitution (replacing ≥51% of fish meal protein) disrupted intestinal morphological integrity. This led to reduced muscularis thickness and fold height, increased lamina propria width, villus breakage, altered gut permeability, and elevated serum endothelin‐1 (ET‐1), D‐lactic acid (D‐LA), 5‐hydroxytryptamine (5‐HT), and diamine oxidase (DAO) levels. These changes are accompanied by changes in the structure of dominant intestinal microflora. Concluded, the proportion of SBM replacing fish meal should be lower than 34% under BFT system.

## 1. Introduction

Owing to its abundance of amino acids, vitamins, and micronutrients, fish meal is a critical high‐quality protein source in formulated fish feeds [1]. However, as intensive aquaculture expands, fish meal resources have become scarce. Consequently, identifying viable alternatives to fish meal protein has become a major focus of aquaculture research. Soybean meal (SBM) is nutrient‐rich, reasonably priced, and widely available. As one of the best plant‐based protein sources, SBM is used in compound fish feeds [2, 3]. However, SBM contains protease (PRS) inhibitors, soybean antigen protein, soybean lectin, and other antinutritional factors (ANFs) [4]. These substances affect fish health by destroying intestinal epithelium, activating inflammatory responses, and interfering with nutrient digestion and absorption [5–7]. Studies involving various species, including rainbow trout (*Oncorhynchus mykiss*) [8], orange‐spotted grouper (*Epinephelus coioides*) [9], Chinese sucker (*Myxocyprinus asiaticus*) [10], Japanese seabass (*Lateolabrax japonicus*) [11], and Asian seabass (*Lates calcarifer*) [12], confirm that excessive substitution of fish meal with SBM adversely affects key physiological processes in aquatic animals, including growth, digestion, metabolism, immunity, and antioxidant defense [13, 14].

At present, reducing the damage to aquatic animals caused by substituting fish meal with SBM and improving the feasibility of such substitution have become new research hotspots. Studies on various species, including rice field eel (*Monopterus albus*) [15], turbot (*Scophthalmus maximus L*.) [16], white seabream (*Diplodus sargus*) [17], largemouth bass (*Micropterus salmoides*) [18], and totoaba (*Totoaba macdonaldi*) [19], have shown that dietary supplementation with sodium butyrate, glutamine, carbohydrases, taurine, and prebiotic agavin can alleviate the detrimental effects on aquatic animals caused by soybean products. Biofloc technology (BFT) is a novel, high‐efficiency ecological aquaculture system [20]. By adding organic carbon sources to adjust the carbon‐to‐nitrogen ratio (C/N) of water, heterotrophic bacteria proliferate and assimilate inorganic nitrogen. Harmful nitrogenous compounds derived from uneaten feed, feces, and metabolites are thus converted into bacterial proteins that can be utilized by cultured animals [21, 22]. In addition, bioflocs can improve water quality, increase feed utilization efficiency, and enhance the immune function in aquatic organisms. Studies in *Ictalurus puntatus* [23], *Cyprinus carpio* [24], *Clarias gariepinus* [25], *Nile tilapia* [26], and *Rhamdia Quelen* [27] have found that bioflocs can enhance growth performance and digestive efficiency, strengthen immune responses, and boost antioxidant capacity in aquatic species. Based on this evidence, we hypothesize that BFT can mitigate the negative impacts associated with high‐level replacement of fish meal with SBM in cultured aquatic species. This is related to the active substances produced during the formation of bioflocs. Polyβ‐hydroxybutyrate (PHB), as one of the main active ingredients (content 5%–20%), can be degraded into β‐hydroxybutyric acid in the intestine and is absorbed and transported to various organs to play its role. Research shows that PHB can promote growth, enhance immunity and antioxidant capacity, and optimize the intestinal flora. Another report pointed out that in the biofloc culture mode, the negative impact of expanded SBM on the intestinal tract of fish has been reduced, which may be related to PHB [28]. Therefore, we speculate that BFT can alleviate the negative impact of high levels of SBM replacing fish meal on farmed aquatic species.

*Rhynchocypris lagowskii* Dybowski is a small omnivorous fish of commercial value in China, known for its delicate flesh, strong disease resistance, and notable tolerance to hypoxic conditions, making it a promising species for aquaculture [29]. Existing studies on this species have addressed its dietary protein requirements [29], tolerance to ANFs (including glycinin threshold [29] and taurine mitigation [30]), and alternative protein sources (SBM and fermented SBM). Therefore, this study aimed to elucidate the effects of replacing fish meal with SBM on the growth, immune function, and gut microbial community of *Rhynchocypris lagowskii* Dybowski under BFT conditions.

## 2. Materials and Methods

### 2.1. Experimental Diets

For BFT, glucose was selected as the carbon source, and the C/N ratio in each group was controlled at 20:1 [29]. The proportions of fish meal protein replaced by SBM were 0.0%, 17.0%, 34.0%, 51.0%, and 68.0%, respectively. These ingredients were used to formulate five isonitrogenous and isolipidic experimental diets, designated as CK, B17, B34, B51, and B68. All feed ingredients were ground, sieved through a 60‐mesh screen, and precisely weighed according to the formulated diet composition (Table 1). After thorough mixing, the mixture was pelleted into 2.0 mm diameter granules, air‐dried, and stored for subsequent use.

**Table 1:** Formulation and nutritional composition of experimental diets (air‐dry basis) (%).

Ingredients	Groups				
	CK	B17	B34	B51	B68
Fish meal^a^	45.00	37.35	29.70	22.05	14.40
Soybean meal^b^	0.00	11.85	23.72	35.58	47.43
Wheatmeal	20.00	20.00	20.00	20.00	20.00
Corn gluten meal	4.00	4.00	4.00	4.00	4.00
Dextrin	22.42	18.25	14.08	9.91	5.75
Cellulose microcrystalline	3.34	2.68	2.01	1.34	0.67
Premix^c^	0.5	0.5	0.5	0.5	0.5
Corn oil	1.78	2.00	2.22	2.44	2.66
Fish oil	1.78	2.00	2.22	2.44	2.66
Choline chloride	0.50	0.50	0.50	0.50	0.50
Lysine	0.24	0.36	0.47	0.59	0.71
L‐methionine	0.44	0.51	0.58	0.65	0.72
Nutritional level^d^	—				
Crude protein	36.09	36.17	36.14	36.20	36.23
Crude lipid	8.13	8.10	8.07	8.03	8.10
Ash	5.93	5.66	5.39	5.12	4.85

^a^Fish meal: 68.00% crude protein and 8.60% crude lipid, from Jingliang (Tianjin) Grain and Oil Industry Co., Ltd., China.

^b^Soybean meal, purchased from Yihai (Taizhou) grain and oil industry co., Ltd., Taizhou, Jiangsu, China. The crude protein content is 44.21% and the crude fat content is 1.70%.

^c^Mineral and vitamin premix (per 100 g premix): vitamin A, 84–104 KIU; vitamin D, 56–59 KUI; vitamin E, ≥2400 mg; vitamin K, ≥162 mg; nicotinamide, ≥458 mg; pantothenic acid, ≥702 mg; folic acid, ≥106 mg; biotin, ≥4 mg; inositol, ≥3020 mg; copper, 96–110 mg; iron, 4060–5500 mg; manganese, 552–633 mg; cobalt, 6–8 mg; iodine, 13–20 mg; zinc, 2320–2660 mg; selenium, 8–12 mg.

^d^Measured value.

### 2.2. Experimental Conditions

*Rhynchocypris lagowskii* Dybowski used in this experiment was purchased from Quanyuan fry farm, Jiutai District, Jilin Province, China. In order to ensure that fish adapt to the experimental feed and laboratory breeding environment more quickly, fish were pre‐fed for 15 days before the start of the feeding experiment. We initiated an 8‐week feeding experiment using 450 healthy juvenile fish (6.89 ± 0.02 g) of uniform size. The fish were distributed among five treatment groups, each comprising three replicates stocked with 30 fish per replicate. During the whole experiment, the daily feeding rate was 2.0%–3.0% of the initial body weight, and it was fed three times a day. Depending on the method published by Yu et al. [29], glucose was selected as the carbon source, and the C/N ratio of each group was controlled to be 20:1. Continuous oxygenation was provided for 24 h and regular replenishment of water lost by evaporation. Glucose powder was added to the fish tank at 18:00 every day and form BFT. All the handling methods in this research are accords with the standards of the Ethics Committee of Jilin Agricultural University.

### 2.3. Fish Sampling

After 56 days of experimental feeding period, all fish were fasted for 24 h and maintained oxygen and carbon source addition (to maintain biological floc activity and water quality stability) to supplement evaporation water and keep water level constant. Initial data collection included counting and weighing all fish per tank to facilitate subsequent growth performance evaluation. The muscles above the lateral line and below the dorsal fin were taken and placed in a refrigerator at −80°C for the determination of muscle nutrients. Blood was drawn from the caudal vein into syringes and aliquoted into 2.0 mL tubes. Following 24 h refrigeration at 4°C, serum was isolated by centrifugation (1400 × *g*, 10 min) for the determination of intestinal permeability and immune markers. Three intestinal segments (proximal intestines [PIs], mid intestines [MIs], distal intestines [DIs]) were collected from 15 sacrificed fish and stored at −80°C for biochemical and molecular analyses. For microbiome studies, intestines from five euthanized fish were processed by removing adhering tissues, rinsing with deionized water, and snap‐freezing in liquid nitrogen before storage at −80°C. For intestinal micromorphological analysis, segments of the PI, MI, and DI were collected from five fish and fixed in 4% formalin solution.

### 2.4. Sample Analysis

#### 2.4.1. Calculations Analysis

The determination of calculation of growth index is based on the method previously described [29].

#### 2.4.2. Determination of Muscle Nutrient Composition

The determination of muscle nutrient content is based on the method previously described [29].

#### 2.4.3. Determination of Levels of Enzymes

A suite of commercial assay kits was employed to quantify multiple biochemical parameters. These included serum biomarkers (diamine oxidase [DAO], PRS, endothelin‐1 [ET‐1], D‐lactic acid [D‐LA], 5‐hydroxytryptamine [5‐HT], lysozyme [LZM], complement 3 [C3], complement 4 [C4], and immunoglobulin M [IgM]), hepatopancreatic and intestinal oxidative stress markers (malondialdehyde [MDA], catalase [CAT], glutathione peroxidase [GSH‐PX], superoxide dismutase [SOD], and total antioxidant capacity [T‐AOC]), as well as digestive and metabolic enzymes (amylase [AMS] and lipase [LPS] in hepatopancreas and intestines; alanine aminotransferase [GPT] and aspartate aminotransferase [GOT] in hepatopancreas and serum). The protein concentration was determined using a protein quantification kit produced by Abbkine (Wuhan) Biotechnology Co., Ltd. For detailed information, please refer to the Supporting Information.

#### 2.4.4. Determination of mRNA Expressions

For the methods, refer to the published articles [31]. Following extraction from hepatopancreatic tissues, total RNA was quantified and assessed for purity. The OD_260_/OD_280_ ratio served as the quality criterion, with values of 1.8–2.0 indicating satisfactory RNA integrity. Following the reverse transcription of RNA into cDNA with a dedicated kit (Takara), gene expression levels were quantified by real‐time PCR employing a fluorescent detection system (Takara). The primer sequences were synthesized by Sangon. The relative expression levels of genes were analyzed by the 2^−ΔΔCT^ method [32] (Table 2).

**Table 2:** Primer sequences of target gene for the real‐time fluorescent quantitative PCR.

Genes	Sequences (5′–3′)		GenBank number
*β-actin*	Forward	CGGTATCCATGAGACCACCT	AAB97964.1
	Reverse	CTTCTGCATCCTGTCAGCAA	
*IGF-1*	Forward	GGAGAAGGAGCGATTGAAGA	AY533140.1
	Reverse	AGGCTTTCCCTTCTCGTCTC	
*mTOR*	Forward	TGCGAATAAGCAGTATGGAGGG	AB290031.1
	Reverse	AAAACTGGTGAAGGGCGTGA	
*S6K*	Forward	GGGCTATGATGAGGG	MK756115.1
	Reverse	CTTCTGGTCCGTTGG	
*4E-BP*	Forward	AACACTCTTCTCCACCAC	KM199776.1
	Reverse	CTCCTCCAACTCCTCTTT	
*NF-κB*	Forward	GAAGAAGGATGTGGGAGATG	KJ526214
	Reverse	TGTTGTCGTAGATGGGCTGAG	
*IL-1*β	Forward	CTGGAGCAATGCAATACAAA	AJ245635
	Reverse	AGGTAGAGGTTGCTGTTGGAA	
*TGF-β*	Forward	TTGGGACTTGTGCTCTAT	EU099588
	Reverse	AGTTCTGCTGGGATGTTT	
*IL-10*	Forward	AATCCCTTTGATTTTGCC	AB110780.1
	Reverse	GTGCCATATCCTACAGTATGTT	
*TNF-α*	Forward	CCAGGCTTTCACTTCAGG	FN543477.1
	Reverse	GCCATAGGAATCGGAGTAG	
*IL-8*	Forward	ATGAGTCTTAGAGGTCTGGGTG	DQ453125
	Reverse	ACAGTGAGGGCTAGGAGGG	
*Nrf2*	Forward	GGAGAAGGAGCGATTGAAGA	MF150102.1
	Reverse	AGGCTTTCCCTTCTCGTCTC	
*Keap1*	Forward	TTCCACGCCCTCCTCAA	XM_019274258.1
	Reverse	TGTACCCTCCCGCTATG	
*HO-1*	Forward	TGTCAGGAGGACAAGTGCTG	JX257180.1
	Reverse	CAGCTGCTTGAATCTGTTGG	
*CAT*	Forward	ACTACCAGTCAACTGCCCGTC	NM_130912.1
	Reverse	TTTAGCACCTGAGTGAAGAACG	
*GSH-PX*	Forward	ACAGAGGGTGGGCGTTAT	AW232474
	Reverse	TTCGGGCACCAGAGGA	
*Maf*	Forward	TGGAGCAGCAGAAGGAG	NM_131844.2
	Reverse	GTGTTCAGACGGGGTGTTGT	
*ZO-1*	Forward	GAAGACAAGCCATACAGA	XM_009303250.3
	Reverse	GTGGGTCAAAGTTACGAG	
*Occludin-a*	Forward	TCCAGCATTTCTACCG	NM_212832.2
	Reverse	ACAGACAAAGATTCCCAC	

*Note: 4E-BP*, eIF 4E binding protein; *S6K*, ribosomal protein S6 kinase, polypeptide.

Abbreviations: *CAT*, catalase; *GSH-PX*, glutathione peroxidase; *HO-1*, heme oxygenase‐1; *IGF-1*, insulin‐like growth factor‐1; *IL-1β*, interleukin‐1β; *IL-8*, interleukin‐8; *IL-10*, interleukin‐10; *Keap1*, kelch‐like ECH‐associated protein 1; *Maf*, musculoaponeurotic fibrosarcoma oncogene; *mTOR*, mammalian target of rapamycin; *NF-κB*, nuclear factor kappa B; *Nrf2*, nuclear factor E2‐related factor; *TGF-β*, transforming growth factor β; *TNF-α*, tumor necrosis factor‐α; *ZO-1*, zonula occludens‐1.

### 2.5. Statistical Analysis

Statistical analyses were performed with SPSS 20.0 using one‐way ANOVA. Significant differences identified by ANOVA were further analyzed with Duncan’s multiple comparison test. Data are expressed as mean ± SE, and differences at *p*  < 0.05 were considered statistically significant.

## 3. Results

### 3.1. Growth and Muscle Nutrient Composition

Under BFT system, compared with the CK group, the B68 group final weight (FW), weight gain rate (WGR), specific growth rate (SGR), feed efficiency ratio (FER), and protein efficiency ratio (PER) were decreased significantly (*p* < 0.05), whereas hepatosomatic index (HSI) and viscerasomatic index (VSI) in other groups show no significant difference (*p* > 0.05) (Table 3).

**Table 3:** Effects of soybean meal replacing fish meal on growth and feed utilization of *Rhynchocypris lagowskii* Dybowski under BFT system.

Indexes	Groups				
	CK	B17	B34	B51	B68
IW (g)	6.87 ± 0.01^a^	6.89 ± 0.15^a^	6.88 ± 0.22^a^	6.93 ± 0.26^a^	6.88 ± 0.24^a^
FW (g)	15.96 ± 0.58^a^	15.84 ± 0.57^a^	15.70 ± 0.64^a^	15.57 ± 0.48^a^	14.07 ± 0.26^b^
WGR (%)	132.37 ± 7.96^a^	130.04 ± 7.47^a^	128.38 ± 9.86^a^	124.60 ± 8.25^a^	104.41 ± 3.81^b^
SGR (%/d)	1.51 ± 0.07^a^	1.49 ± 0.06^a^	1.47 ± 0.08^a^	1.44 ± 0.07^a^	1.28 ± 0.04^b^
FER (%)	70.48 ± 4.63^a^	69.13 ± 4.78^a^	66.17 ± 4.98^a^	63.66 ± 2.54^a^	54.52 ± 4.23^b^
PER (%)	195.78 ± 12.87^a^	192.04 ± 13.30^a^	183.81 ± 13.83^a^	176.83 ± 7.06^a^	151.43 ± 11.74^b^
CF (g/cm^3^)	2.03 ± 0.52^a^	2.40 ± 0.23^a^	2.24 ± 0.16^a^	2.31 ± 0.10^a^	2.15 ± 0.14^a^
VSI (%)	11.63 ± 1.27^a^	11.36 ± 0.46^a^	10.66 ± 0.61^a^	10.62 ± 1.66^a^	10.91 ± 1.21^a^
HSI (%)	2.35 ± 0.15^a^	2.15 ± 0.13^a^	2.23 ± 0.33^a^	2.29 ± 0.16^a^	2.14 ± 0.12^a^
SR (%)	92.22 ± 3.85^a^	93.34 ± 6.67^a^	97.78 ± 1.92^a^	94.44 ± 5.09^a^	94.67 ± 4.33^a^

*Note:* Different superscript lowercase letters on the shoulder of the same data indicate significant differences (*p* < 0.05).

Abbreviations: CF, condition factor; FER, feed efficiency ratio; FW, final weight; HSI, hepatosomatic index; IW, initial weight; PER, protein efficiency ratio; SGR, specific growth rate; SR, survival rate; VSI, viscerasomatic index; WGR, weight gain rate.

### 3.2. Muscle Nutrients

Muscle crude protein content was significantly lower in the B68 group than in the CK group (*p* < 0.05), whereas no significant differences were observed in muscle moisture, crude lipid, or ash content among groups (*p* > 0.05) (Table 4).

**Table 4:** Effects of soybean meal replacing fish meal on muscle nutrients of *Rhynchocypris lagowskii* Dybowski under BFT system.

Groups	Indexes (%)			
	Moisture	Crude lipid	Ash	Crude protein
CK	77.17 ± 0.71^a^	1.28 ± 0.28^a^	1.48 ± 0.06^a^	18.05 ± 0.31^a^
B17	77.42 ± 0.45^a^	1.39 ± 0.15^a^	1.40 ± 0.08^a^	17.80 ± 0.07^ab^
B34	77.20 ± 0.47^a^	1.38 ± 0.26^a^	1.45 ± 0.21^a^	17.82 ± 0.41^ab^
B51	76.99 ± 0.50^a^	1.37 ± 0.19^a^	1.30 ± 0.08^a^	17.48 ± 1.22^ab^
B68	77.30 ± 0.80^a^	1.41 ± 0.09^a^	1.42 ± 0.18^a^	16.70 ± 0.71^b^

*Note:* Different superscript lowercase letters on the shoulder of the same data indicate significant differences (*p* < 0.05).

### 3.3. Digestive Enzyme Activities

**Figure 1:**
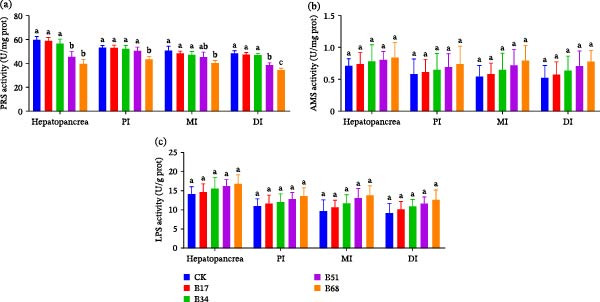
Effects of soybean meal substitution for fishmeal on the intestinal digestive enzymes activities in *Rynchocypris lagowskii* Dybowski under BFT system. (a): PRS, protease; (b): AMS, amylase; (c): LPS, lipase;. Different lowercase letters above the bars show significant differences (*p* < 0.05).

Relative to the CK group, the B68 group exhibited significantly reduced PRS activities in the hepatopancreas and all intestinal segments (PI, MI, and DI) (*p* < 0.05). In contrast, the substitution of fish meal with SBM did not significantly alter the activities of AMS and LPS in these tissues (*p* > 0.05) (Figure 1).

### 3.4. Protein Metabolism

**Figure 2:**
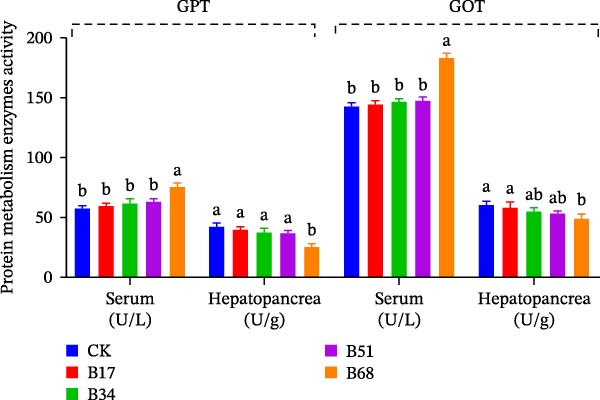
Effects of replacing fishmeal with soybean meal on the protein metabolism activities of the *Rhynchocypris lagowskii* Dybowski under BFT system. GOT, aspartate aminotransferase; GPT, alanine aminotransferase. Different lowercase letters above the bars show significant differences (*p* < 0.05).

**Figure 3:**
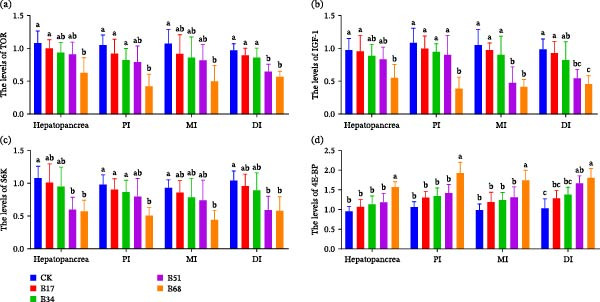
Effects of soybean meal substitution for fishmeal on the mRNA expression levels related to protein metabolism in *Rlynchocypris lagowskii* Dybowski under BFT system. (a): mTOR, mechanistic target of rapamycin; (b): IGF‐1, insulin‐like growth factor 1; (c): S6K, ribosomal protein S6 kinase; (d): 4E‐BP, eIF4E‐binding protein. Different lowercase letters above the barsshow significant differences (*p* < 0.05).

Significant metabolic alterations were observed in treatment groups. The B68 group showed increased serum GPT/GOT with decreased hepatopancreatic activities (*p* < 0.05), plus comprehensive downregulation of *mTOR*, *IGF-1*, and *S6K* transcripts and *4E-BP* upregulation across all tissues. The B51 group exhibited targeted gene expression changes: reduced *mTOR* (DI), *IGF-1* (MI and DI), and *S6K* (hepatopancreas and DI) with elevated *4E-BP* (DI) (*p* < 0.05) (Figures 2 and 3).

### 3.5. Immune Function and Inflammation

**Figure 4:**
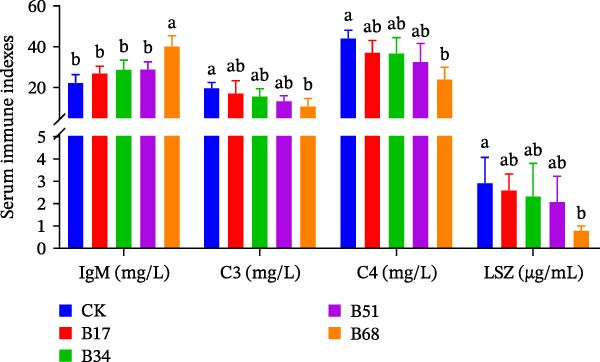
Effects of soybean meal substitution for fishmeal on the immune function in *Rhynchocypris lagowskii* Dybowski under BFT system. C3, complement 3; C4, complement 4; IL‐1β: interleukin‐1 beta; IL‐8: interleukin‐8; IL‐10: interleukin‐10; NF‐κB, nuclear factor kappa‐light‐chain‐enhancer of activated B cells; TGF‐β: transforming growth factor beta; TNF‐α, tumor necrosis factor alpha. Different lowercase letters above the bars show significant differences (*p* < 0.05).

**Figure 5:**
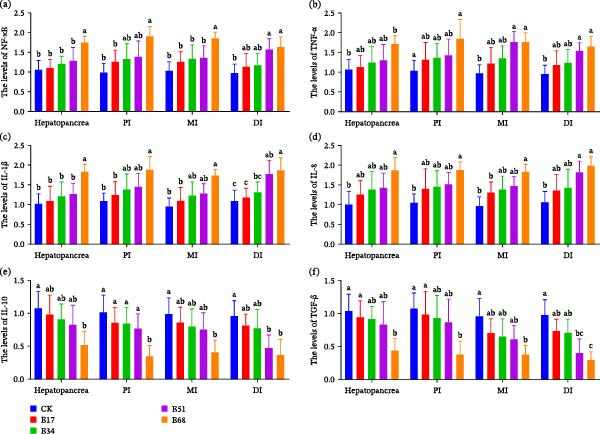
Effects of soybean meal substitution for fishmeal on the expression levels of immune‐related mRNA in *Rhynchocypris lagowskii* Dybowski under BFT system. (a): NF‐kB, nuclear factor kappa‐light‐chain‐enhancer of activated B cells; (b): TNF‐a, tumor necrosis factor alpha; (c): IL‐1β: interleukin‐1 beta; (d): IL‐8: interleukin‐8; (e): IL‐10: interleukin‐10; (f): TGF‐β: transforming growth factor beta. Different lowercase letters above the bars show significantdifferences (*p* < 0.05).

Significant decreases in serum C3, C4, and LSZ activity, along with an increase in IgM, were observed in the B68 group compared to the control group (*p* < 0.05). Relative to the control, mRNA levels of *NF-κB*, *IL-1β*, and *IL-8* were significantly upregulated in the hepatopancreas, PI, MI, and DI of the B68 group and in the DI of the B51 group (*p* < 0.05). In contrast, *TGF-β* mRNA was downregulated in these same tissues (*p* < 0.05). Additionally, *TNF-α* mRNA was significantly increased in the hepatopancreas, PI, MI, and DI of the B68 group, as well as in the MI and DI of the B51 group (*p* < 0.05) (Figures 4 and 5).

### 3.6. Antioxidant Capacity

**Figure 6:**
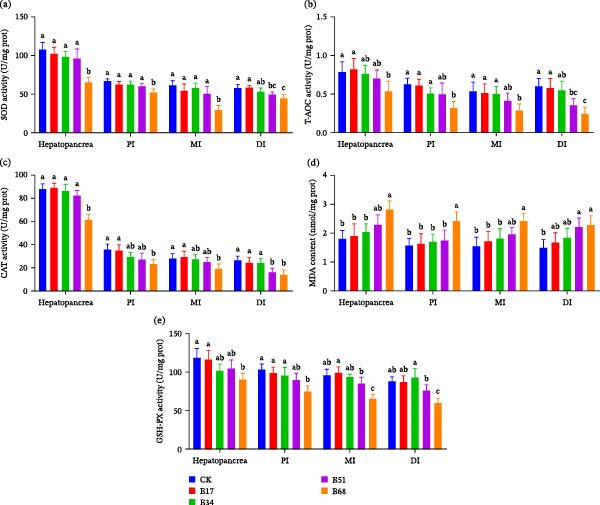
Effects of soybean meal substitution for fishmeal on the antioxidant indexes in *Rlynchocypris lagowskii* Dybowski under BFT system. (a): T‐SOD, totalsuperoxide dismutase; (b): T‐AOC, total antioxidant capacity; (c): CAT, catalase; (d): MDA, malondialdehyde; (e): GSH‐Px, glutathioneperoxidase. Different lowercase letters above the bars show significant differences (*p* < 0.05).

**Figure 7:**
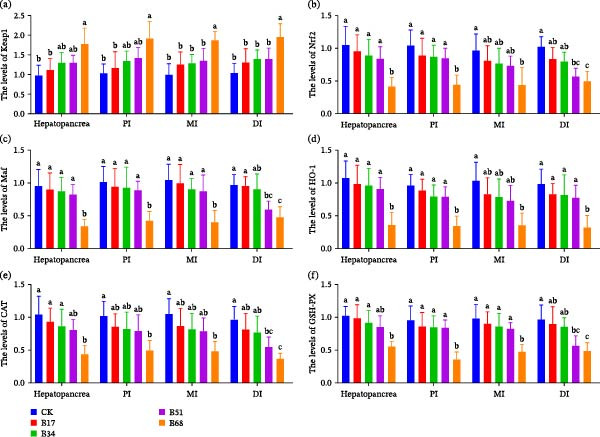
Effects of soybean meal substitution for fishmeal on the the expression levels of antioxidant‐related mRNA in *Rhynchocypris lagowskii* Dybowski under BFT system. (a): Keapl, kelch‐like ECH‐associated protein1; (b): Nrf2, nuclear factor erythroid 2‐related factor 2; (c): Maf, musculoaponeurotic fibrosarcomaoncogene homolog; (d): HO‐1, heme oxygenase‐1; (e): CAT, catalase; (f):GSH‐Px, glutathioneperoxidase. Different lowercase letters above the bars show significant differences (*p* < 0.05).

Relative to the control, significant reductions in the activities of SOD, CAT, T‐AOC, and GSH‐PX were observed in the hepatopancreas, PI, MI, and DI of the B68 group and in the DI of the B51 group, concurrently with an increase in MDA content in these same tissues (*p* < 0.05). At the transcriptional level, mRNA levels of *Nrf2*, *Maf*, *CAT*, *HO-1*, and *GSH-PX* were downregulated in the hepatopancreas, PI, MI, and DI of the B68 group and in the DI of the B51 group (*p* < 0.05). In contrast, *Keap1* mRNA was significantly upregulated in the hepatopancreas, PI, MI, and DI of the B68 group (*p* < 0.05) (Figures 6 and 7).

### 3.7. Intestinal Physical Barrier

**Figure 8:**
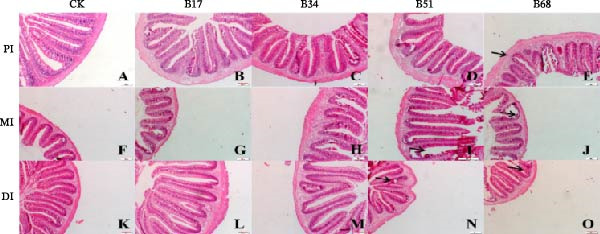
Effects of soybean meal substitution for fishmeal on the intestinal morphology in *Rlynchocypris lagowskii* Dybowski under BFT system (200x). PI: proximal intestine; MI: middle intestine; DI: distal intestine. No additional explanation is needed for the other annotations in this figure.

Under the biofloc culture regime, the intestinal morphology was significantly altered by high levels of SBM substitution, and histopathological examination revealed that the B51 group displayed villus atrophy and breakage in the midgut, whereas the hindgut showed additional villus atrophy and lamina propria separation (Figure 8).

Groups B68 (all intestinal segments) and B51 (DI only) displayed significantly reduced intestinal fold height and muscle layer thickness, mirrored by a significant widening of the lamina propria in these same segments (*p* < 0.05). Intestinal mass, length, and related indices remained unchanged across all groups (Table 5).

**Table 5:** Effects of soybean meal replacing fish meal on the intestinal morphological indicators of *Rhynchocypris lagowskii* Dybowski under BFT system

Indexes	Groups				
	CK	B17	B34	B51	B68
Intestinal weight (g)	0.29 ± 0.10^a^	0.27 ± 0.06^a^	0.28 ± 0.10^a^	0.28 ± 0.03^a^	0.27 ± 0.07^a^
Intestinal length (cm)	6.79 ± 0.11^a^	6.73 ± 0.29^a^	6.65 ± 0.23^a^	6.51 ± 0.17^a^	6.44 ± 0.11^a^
Intestinal somatic index (%)	4.98 ± 0.43^a^	5.08 ± 0.52^a^	5.24 ± 0.74^a^	5.39 ± 0.48^a^	5.41 ± 0.22^a^
Intestinal length index (%)	0.92 ± 0.10^a^	0.95 ± 0.10^a^	0.95 ± 0.09^a^	0.95 ± 0.07^a^	0.98 ± 0.16^a^
PI folds heights (µm)	672.10 ± 51.83^a^	625.79 ± 22.70^a^	581.87 ± 48.45^ab^	581.68 ± 55.09^ab^	521.57 ± 74.00^b^
MI folds heights (µm)	472.74 ± 73.43^a^	419.39 ± 6.72^a^	387.29 ± 62.71^ab^	371.21 ± 62.59^ab^	290.29 ± 51.85^b^
DI folds heights (µm)	352.23 ± 48.36^a^	314.24 ± 57.26^ab^	288.70 ± 27.13^ab^	247.93 ± 41.00^bc^	187.45 ± 31.09^c^
PI muscular layer thickness (µm)	20.09 ± 2.05^a^	18.21 ± 2.21^a^	16.98 ± 2.06^a^	15.93 ± 3.90^a^	10.68 ± 3.31^b^
MI muscular layer thickness (µm)	17.53 ± 3.32^a^	16.31 ± 3.56^a^	14.65 ± 3.52^ab^	12.43 ± 4.06^ab^	8.11 ± 3.64^b^
DI muscular layer thickness (µm)	15.27 ± 3.30^a^	13.95 ± 3.29^ab^	13.43 ± 2.50^ab^	8.29 ± 3.79^b^	7.75 ± 3.59^b^
PI lamina propria width (µm)	14.66 ± 3.50^a^	15.07 ± 3.02^a^	16.79 ± 3.74^ab^	17.07 ± 3.05^ab^	22.95 ± 3.68^b^
MI lamina propria width (µm)	12.25 ± 3.28^a^	13.62 ± 3.50^a^	14.40 ± 3.39^a^	15.84 ± 3.54^ab^	20.95 ± 3.08^b^
DI lamina propria width (µm)	9.51 ± 1.27^a^	11.80 ± 3.51^a^	12.28 ± 3.90^a^	19.44 ± 3.87^b^	19.64 ± 3.69^b^

*Note:* Different superscript lowercase letters indicate significant differences (*p* < 0.05).

Disruption of intestinal morphological structure may be accompanied by alterations in intestinal permeability and tight junction (TJ) mRNA expression. Through the detection of relevant indicators, it was found that the ET‐1, D‐LA, and 5‐HT contents and DAO activities in serum of B68 group and B51 group were significantly increased (*p* < 0.05) (Table 6).

**Table 6:** Effects of soybean meal replacing fish meal on intestinal permeability of *Rhynchocypris lagowskii* Dybowski under BFT system.

Groups	ET‐1 (pg/mL)	D‐LA (μmol/L)	5‐HT (ng/mL)	DAO (U/mL)
CK	17.55 ± 1.74^a^	11.63 ± 1.43^a^	22.48 ± 2.06^a^	28.67 ± 1.49^a^
B17	18.86 ± 1.51^a^	12.20 ± 1.51^a^	23.02 ± 1.63^a^	30.24 ± 1.99^a^
B34	19.62 ± 0.98^a^	13.57 ± 2.30^a^	24.38 ± 1.86^a^	31.61 ± 1.74^a^
B51	20.13 ± 2.18^a^	14.25 ± 1.85^a^	25.13 ± 1.20^ab^	37.82 ± 2.11^b^
B68	24.21 ± 1.53^b^	19.12 ± 2.31^b^	27.89 ± 0.25^b^	38.83 ± 2.51^b^

*Note:* Different superscript lowercase letters indicate significant differences (*p* < 0.05).

**Figure 9:**
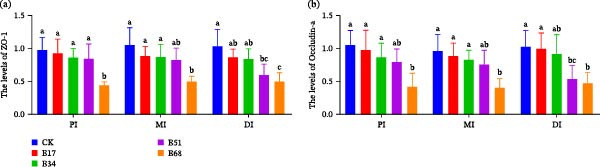
Effects of soybean meal substitution for fishmeal on the intestinal tight junction‐associated mRNA expression levels in *Rlynchocypris lagowskii* Dybowski under BF’Isystem. (a): ZO‐1; (b): Occludin‐a. Different lowercase letters above the bars show significant differences (*p* < 0.05).

The levels of *ZO-1* and *occludin-a* mRNA in intestinal canal of B68 group and DI in B51 group were significantly decreased (*p* < 0.05) (Figure 9).

### 3.8. Intestinal Microbiota

**Figure 10:**
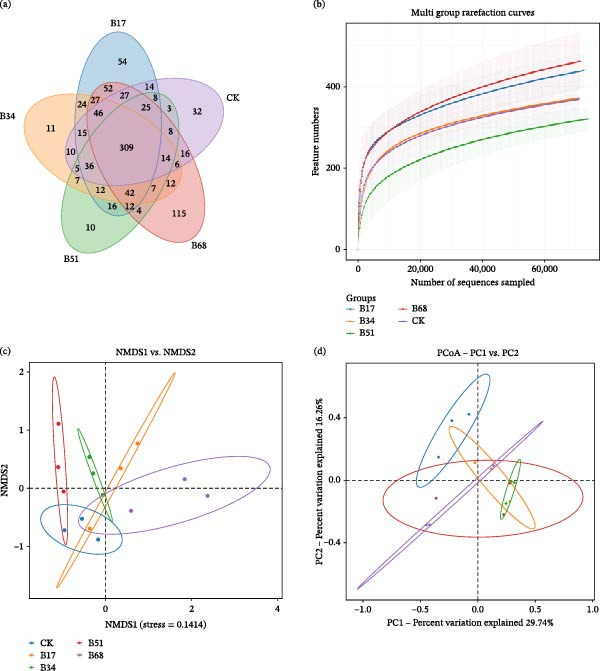
Effects of soybean meal substitution for fishmeal on the analysis of α diversity in intestinal flora in *Rhynchocypris lagowskii* Dybowski under BFT system. (a) Venn map of OUT by high‐throughput sequencing, (b) dilution curve of intestinal flora (c) NMDS analysis of intestinal flora, and (d) PCoA analysis of intestinal flora.

According to statistics, a total of 1,199,905 sequenced reads were obtained from 15 samples. After clustering OTUs with 97% consistency, a total of 979 OUTs were obtained. The sparse curve shows that the sampling depth of this test meets the requirements, and the sequencing results can truly reflect the dominant flora in the sample (Figure 10a). The β‐diversity analysis showed that the distance between the replacement group and the control group was relatively close but clearly separated, indicating that there was a significant difference in the composition of the intestinal microbiota between the replacement group and the control group. The replacement groups clustered adjacently in a gradient‐like pattern, indicating that the effect of SBM substitution for fishmeal on the intestinal microbiota of *Rhynchocypris lagowskii* Dybowski larvae changed gradually with the substitution ratio (Figure 10b–d).

The α‐diversity analysis showed that Simpson in B51 group were significantly decreased (*p* < 0.05), Shannon in B68 group were significantly increased (*p* < 0.05), however, the replacement of fish meal by SBM had no significant effect on ACE and Chao1 (*p* > 0.05) (Table 7).

**Table 7:** The α‐diversity of intestinal microflora in *Rhynchocypris lagowskii* Dybowski.

Groups	Indexes				
	OTUs	ACE indexes	Chao1 indexes	Simpson indexes	Shannon indexes
CK	372.00 ± 48.78^ab^	501.74 ± 131.16^a^	484.33 ± 105.45^ab^	0.87 ± 0.12^ab^	4.83 ± 0.63^ab^
B17	442.00 ± 38.97^bc^	677.99 ± 159.06^a^	577.33 ± 50.74^b^	0.84 ± 0.16^abc^	4.93 ± 1.09^ab^
B34	375.33 ± 8.39^ab^	548.69 ± 133.47^a^	489.12 ± 30.62^ab^	0.73 ± 0.07^bc^	3.70 ± 0.27^a^
B51	322.00 ± 29.46^a^	482.59 ± 82.56^a^	430.42 ± 31.26^a^	0.65 ± 0.11^c^	2.88 ± 0.93^a^
B68	463.67 ± 68.13^c^	693.47 ± 91.93^a^	597.67 ± 60.29^b^	0.96 ± 0.05^a^	6.55 ± 1.84^b^

*Note:* Different superscript lowercase letters indicate significant differences (*p* < 0.05).

**Figure 11:**
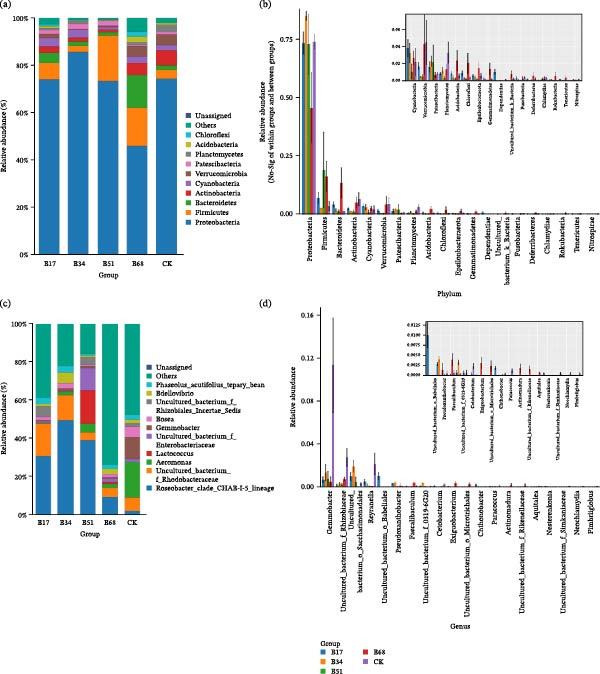
Effects of soybean meal replacing fish meal on composition and abundance of intestinal flora of *Rhynchocypris lagowskii* Dybowski under BFT system. (a, b) Phylum level. (c, d) Genus level.

At the phylum level, the dominant phylum was *Proteobacteria*, *Firmicutes*, *Bacteroidetes*, *Actinobacteria*, and *Cyanobacteria*. *Proteobacteria* dominated in the intestinal flora of fish. The abundance of *Proteobacteria* in B68 group was significantly lower than control group (*p* < 0.05). At the genus level, the top five species with relative abundance were Roseobacter clade CHAB‐1–5 lineage, uncultured‐bacterium f Rhodobacteraceae, *Aeromonas*, *Lactococcus*, and uncultured bacterium f Enterobacteriaceae. *Gemmobacter* and uncultured bacterium f Rhizobiales Incertae Sedis were dominant in the intestinal flora of fish. The abundance of *Gemmobacter* and uncultured bacterium f Rhizobiales Incertae Sedis of B68 group were significantly lower than control group (*p* < 0.05) (Figure 11).

## 4. Discussion

Fish growth, achieved through feed ingestion and metabolic conversion into somatic tissue [28, 33], is impaired in BFT systems when SBM replaces more than 51% of fish meal. This impairment results from the high replacement level combined with SBM‐related challenges, including ANFs and an imbalanced amino acid profile, which ultimately compromise growth performance [34]. Evidence from diverse species—Japanese seabass (*Lateolabrax japonicus*) [11], cobia (*Rachycentron canadum*) [4], amberjack (*Seriola dumerili*) [35], spotted rose snapper (*Lutjanus guttatus*) [36], and tilapia (*Oreochromis niloticus*) [37]—confirms that elevated SBM inclusion consistently suppresses the growth of aquatic animals. Compared with conventional systems, BFT markedly increases the suitable SBM substitution level for fish meal in feed for *Rhynchocypris lagowskii* [29]. This enhancement presumably results from biofloc ingestion under optimized carbon‐nitrogen ratios in BFT, which robustly stimulates growth and maximizes production outcomes [24, 38, 39]. Concurrently, fish body composition provides critical insights into the nutritional status and physiological health [40]. In the BFT system, the significant decrease in muscle crude protein content observed in the B68 group compared to the CK group suggests that high‐level SBM substitution inhibits protein deposition in fish. This effect may stem from an amino acid imbalance and structural alterations in the digestive tract induced by SBM, ultimately impairing protein synthesis [41]. Consistent with this, studies on Japanese seabass [11], amberjack [35], tin foil barb [42], and rainbow trout [8] have reported reduced muscle crude protein following high SBM inclusion in compound feed. Notably, in the present BFT system, bioflocs—rich in nutrients and probiotics—could partially compensate for nutritional requirements and mitigate ANF‐related damage [43], though this did not fully counteract the negative impact of excessive SBM replacement on protein deposition.

The activities of core digestive enzymes—AMS, PRS, and LPS—govern nutrient digestion in aquatic animals, profoundly shaping their digestive efficiency [11, 44]. PRS, essential for dietary protein hydrolysis, is a recognized biomarker of protein utilization ability in fish [45]. In this BFT‐based study, significant declines in hepatopancreatic and intestinal PRS activity were recorded in B51 and B68 groups compared to controls. This response may be linked to ANFs in SBM, which, at high inclusion levels, can disrupt digestive processes and suppress enzyme function [46]. Some studies have shown that increasing SBM in feed can reduce the PRS activity in hepatopancreas, as described by the results obtained in tilapia (*Oreochromis niloticus* × *O. aureus*) [47], red seabream (*Pagrus major*) [48], gibel carp (*Carassius auratus gibelio*) [49], and Amur Sturgeon (*Acipenser schrenckii*) [50]. However, under the BFT system, SBM instead of fish meal has no significant effect on activities of AMS and LPS. Similar results were found in amberjack [35] and abalone (*Haliotis discus hannaino*) [51]. The consistent starch and lipid profiles in all diets likely explain the unaltered LPS and AMS activities observed after SBM replacement in the BFT system since these enzymes respond primarily to their specific substrates. Alternatively, the modulations in fish intestinal digestive enzyme activity noted in this study may be linked to biofloc inclusion, given its recognized constituents—polysaccharides and extracellular enzymes—that enhance digestive enzyme function in farmed animals [29, 51–54]. As biomarkers of protein metabolism, GPT and GOT activities reflect an organism’s capacity for protein turnover [28]. We observed significantly raised serum levels and reduced hepatopancreatic activities of both enzymes in the B68 group versus CK, indicative of SBM‐related hepatopancreatic injury that disrupts normal transaminase distribution [55]. These results align with reports in tilapia [37], obscure puffer [56], Japanese seabass [57], and abalone [51], collectively highlighting a common physiological response to high SBM diets in aquatic species. These findings likely reflect biofloc‐mediated effects, attributable to the presence of PHB‐synthesizing bacteria in bioflocs. When ingested, these bacteria improve intestinal peristalsis and upregulate proteolytic enzyme activity, facilitating nutrient assimilation and metabolism [58]. Meanwhile, the mTOR pathway coordinates cellular growth in response to nutritional cues by activating anabolic processes (e.g., protein and nucleic acid synthesis) and inhibiting autophagy [30]. Under BFT conditions, the B68 group exhibited markedly reduced mRNA expression of *mTOR*, *IGF-1*, and *S6K*, together with elevated *4E-BP* levels in the DI compared to the control. This pattern is consistent with the expression pattern of genes related to protein metabolism inhibition, suggesting that there may be a molecular basis for protein synthesis downregulation. An imbalanced amino acid composition resulting from high SBM substitution likely underlies the impaired protein utilization and synthesis efficiency [59]. Consistent with this, hepatopancreatic IGF‐1 expression was reduced in multiple aquatic species fed high plant‐protein diets [60]. While S6K1 was upregulated and 4E‐BP1 downregulated in Chinese mitten crab hepatopancreas [53], biofloc‐derived nutrients and beneficial microbes in the present system may help mitigate such disruptions, thereby enhancing protein turnover in *Rhynchocypris lagowskii* Dybowski [61].

Fish rely predominantly on nonspecific immunity due to their incomplete specific immune mechanisms compared to those of mammals [62, 63]. In this BFT study, the B68 group showed suppressed C3, C4, and LSZ but elevated IgM versus controls, indicating that SBM substitution within an appropriate range enhances immunity, whereas excessive replacement causes intestinal impairment and immune dysregulation. Immune suppression in fish fed high SBM diets is likely attributable to ANFs and suboptimal amino acid profiles, which collectively undermine nutritional adequacy [5]. Similar findings have been reported in diverse species such as Pacific white shrimp [64] and various finfish [65]. These results corroborate earlier work in *Rhynchocypris lagowskii* Dybowski, confirming that excessive SBM inclusion compromises immunity [29]. The results demonstrate that a suitable carbon‐nitrogen ratio strengthens immune responses in aquatic organisms. This enhancement may be driven by biofloc components—including diverse microorganisms, extracellular enzymes, and probiotics—that upregulate immune enzyme activity and produce bioactive secondary metabolites [66]. In parallel, plant protein‐induced regulation of intestinal inflammatory cytokines is widely recognized to involve the transcription factor NF‐κB [67]. These cytokines are functionally divided into pro‐inflammatory and anti‐inflammatory groups according to their specific roles in immune responses. Relative to controls in the BFT system, the B68 group displayed upregulated NF‐κB, IL‐1β, IL‐8, and downregulated TGF‐β mRNA throughout the hepatopancreas and intestinal tract, while the B51 group showed elevated NF‐κB, IL‐1β, IL‐8, and TNF‐α with reduced TGF‐β limited to the DI. These gene expression changes suggest that SBM‐associated ANFs can induce immune dysfunction, ultimately hindering normal growth in fish [28]. These gene expression changes suggest that SBM‐associated ANFs may induce immune dysfunction and ultimately impede normal fish growth [28]. SBM substitution in Japanese seabass diets enhanced pro‐inflammatory mediators (TNF‐α, IL‐1β, and IL‐8) and reduced anti‐inflammatory gene expression [11]. Parallel observations in rainbow trout indicated that high SBM intake provoked intestinal inflammation and significantly raised IL‐1β, IL‐10, and IL‐17 transcript levels [68]. These results are consistent with our data. The mitigation of inflammation in the current study could be due to high C/N bioflocs, which harbor beneficial microorganisms, probiotics, and bioactive agents that potentiate immune function and inflammatory control in aquatic species [24, 69, 70].

Inflammation and oxidative stress are fundamentally interconnected [71]. Biological organisms require oxygen‐free radicals for normal physiological functions but rely on both enzymatic and non‐enzymatic antioxidant systems to maintain redox balance. A shift of this dynamic equilibrium toward oxidation results in oxidative stress damage [72]. Under BFT conditions, the B68 group showed markedly decreased SOD, CAT, T‐AOC, and GSH‐PX activities and increased MDA levels in the hepatopancreas and entire intestinal tract, with the B51 group exhibiting similar trends specifically in the DI. Consistent with observations in numerous aquatic animals [73], soybean‐derived materials reduced antioxidant potential. In this study, the increase of MDA in the B68 group was accompanied by the decrease of SOD, CAT, and GSH‐PX activities, forming a coupled model of oxidative stress markers, which confirmed that excessive replacement of SBM destroyed the dynamic balance between antioxidant defense and reactive oxygen species (ROS) production. This state of oxidative stress may result from ANFs‐induced inflammation in SBM that produces excess ROS that impair immune pathways and impair antioxidant function [74]. The present results align with previous findings from clear‐water culture systems, wherein an increased substitution of fish meal by SBM was implemented [29]. This consistency may be attributed to the ability of BFT to enhance the antioxidant status of aquatic organisms, attenuating their sensitivity to oxidative stress and thereby strengthening their antioxidant capacity [75]. Notably, the Nrf2‐Keap1/ARE signaling pathway represents a canonical regulatory axis for modulating the antioxidant defense system. It can be activated under exogenous oxidative stress, helping maintain intracellular redox homeostasis and reducing cellular susceptibility to apoptotic signals [76, 77]. In the BFT system, the B68 group exhibited suppressed mRNA expression of Nrf2, Maf, and downstream antioxidant genes (CAT, HO‐1, and GSH‐PX) in the hepatopancreas and entire intestinal tract, along with elevated Keap1 transcript levels; similar alterations occurred in the DI of the B51 group. These results suggest that SBM impairs antioxidant capacity in fish via the Keap1‐Nrf2‐ARE signaling axis, which may be initiated by ANFs in SBM, which promote inflammatory response and excessive production of ROS, leading to oxidative cell damage and gene expression disorder in the Keap1‐Nrf2‐ARE cascade, ultimately impairing antioxidant response [78]. Consistent observations have been documented in multiple aquatic species, including *Rhynchocypris lagowskii* Dybowski [79], pearl gentian groupers (*Epinephelus fuscoguttatus♀ × Epinephelus lanceolatus♂*) [80], and *Channa argus* [81]. In the present study, BFT effectively mitigated the adverse effects induced by SBM [29]. This protective role may be attributed to the diverse microbial communities in BFT systems, where both the microorganisms and their metabolites serve as immunostimulatory agents that enhance the antioxidant capacity in aquatic organisms [82, 83].

As the primary site for nutrient digestion and absorption, intestinal structural integrity is crucial for the healthy development of aquatic animals [28]. Under BFT conditions, the B68 group exhibited significantly reduced fold heights and muscular layer thickness in the PI, MI, and DI, along with increased lamina propria width in these segments. The B51 group showed similar alterations, though confined to the DI. These morphological changes suggest that ANFs in SBM—including β‐conglycinin, lectin, and soybean antigens—induce intestinal inflammation, impair normal intestinal development, and cause histopathological damage in *Rhynchocypris lagowskii* Dybowski [11]. Consistent results have been reported in *Lateolabrax japonicus* [11] and *Rhynchocypris lagowskii* Dybowski. Multiple ANFs in SBM may damage the gut through synergistic effects: trypsin inhibitors reduce protein digestibility, resulting in fermentation of undigested proteins in the hindgut to produce harmful substances; soy antigenic proteins (β‐conglycinin/glycinin) activate intestinal immune response and disrupt TJs; and phytic acid chelates minerals and affects enzyme activity [84]. The decrease in PRS activity in this study (group B68) suggests that trypsin inhibitors may play a major role, while the extensive upregulation of inflammatory factors suggests that the immune activation effect of antigenic proteins is significant. The mitigation of such damage in BFT systems may be attributed to growth‐promoting and bioactive compounds in bioflocs, which under suitable C/N ratios allow higher SBM inclusion while maintaining optimal growth performance [85]. The increased intestinal permeability in *Rhynchocypris lagowskii* Dybowski likely results from the disruption of intestinal TJ structures by ANFs—including trypsin inhibitors, phytic acid, soybean lectin, oligosaccharides, antigenic proteins, and urease—present in SBM used to replace fish meal. TJs, composed of proteins such as occludin and claudins, play a critical role in regulating paracellular permeability [86]. Consistent with this, the B68 group exhibited significantly elevated serum levels of ET‐1, D‐LA, 5‐HT, and DAO activity, while the B51 group showed increased DAO activity. Concurrently, mRNA expression of ZO‐1 and occludin‐a was markedly downregulated in the intestinal tract of the B68 group and in the DI of the B51 group. This may be because the gastrointestinal tract is the main target of SBM‐induced alterations in *Rhynchocypris lagowskii* Dybowski. Pathological changes are primarily driven by ANFs (soybean globulin, β‐conglycinin, etc.). that impair digestive function and initiate detrimental physiological cascades, ultimately affecting growth and gut health [6]. The biofloc system supports higher SBM substitution compared to conventional aquaculture by serving as a source of supplemental nutrition, probiotics, and bioactive substances, with optimal C/N ratios further enhancing intestinal well‐being [87].

Under the biofloc culture model, the substitution of fishmeal with SBM significantly altered the gut microbiota structure and diversity in Phoxinus lagowskii [28]. Specific manifestations included a marked reduction in the abundance of Proteobacteria in the B68 group, a decrease in the Simpson index in the B51 group, and an increase in the Shannon index in the B68 group. Beta‐diversity analysis further confirmed a clear separation between the substitution groups and the control group, with gradient shifts indicating a dose‐dependent restructuring of the gut microbiota. These findings suggest that SBM substitution may drive microbial restructuring by modifying the intestinal microenvironment. Multiple studies indicate that the impact of SBM on the fish microbiota is species‐ and condition‐dependent. Research by Tan et al. [88] collectively reports that SBM can increase microbial diversity and alter phylum‐level composition. In contrast, the results of Reveco, Desai, and Bruce show inconsistencies, highlighting the complexity of SBM’s effects on the gut microbiota [89–91]. This divergence may stem from interactions among variables such as the inclusion level of SBM, trial duration, and rearing environment. Notably, although the biofloc system can support higher substitution ratios of SBM, its mitigating effect may have an upper limit [87]. The unique ANFs and nutritional composition of SBM are likely to reshape the metabolic landscape of the gut microbiota, promoting the proliferation of specific bacterial taxa and thereby breaking the pre‐existing ecological balance [28]. Thus, the observed structural shifts in the microbiota are not merely a direct outcome of SBM substitution but also a potential mechanism underlying its impact on the host health.

## 5. Conclusion

In the BFT system, in order to guarantee growth performance and intestinal health, the proportion of SBM replacing fishmeal should be controlled below 34%, the maximum tolerable replacement ratio should be 51%, replacing ≥51% of fish meal protein with SBM significantly suppressed the growth performance of juvenile *R. lagowskii*, impaired intestinal morphology and barrier function, induced oxidative stress and inflammation, and caused intestinal dysbiosis. Therefore, the substitution level should be kept below 51%.

## Author Contributions

**Zhi-Yong Yang:** writing – original draft, validation, methodology. **Deng-Lai Li:** methodology, investigation, formal analysis. **Ke-xin Wang:** validation, methodology. **Rui Zhu:** validation, investigation. **Li-Fang Wu:** supervision, resources, project administration. **Qiong Wu:** supervision, conceptualization.

## Funding

The work was supported by the Jilin Provincial Scientific and Technological Development Program (Grant 20200402038NC).

## Disclosure

This manuscript was approved for publication by all authors.

## Conflicts of Interest

The authors declare no conflicts of interest.

## Supporting Information

Additional supporting information can be found online in the Supporting Information section.

## Supporting information


**Supporting Information** The supporting information associated with this manuscript provides detailed information about the commercial kits used in the enzyme activity assays described in Section 2.4.3. Specifically, it includes the manufacturer, catalog numbers, and the detection principles for each kit employed to measure the target enzyme activities. This supporting information is intended to support the reproducibility of the experimental procedures. Please refer to the supporting information document for full details.

## Data Availability

Data will be provided upon request.
