# Supplementary material for: A Maximum 34% Substitution of Fish Meal by Soybean Meal Is Recommended for Rhynchocypris lagowskii Dybowski Cultured in Biofloc Systems: Evidence From Growth and Intestinal Barrier Function
Source: Aquac Nutr. 2026 Jul 28;2026:5561983. doi: 10.1155/anu/5561983 (PMC13409043; doi:10.1155/anu/5561983)
Supplement: Supplementary file 1 — Supporting Information The supporting information associated with this manuscript provides detailed information about the commercial kits used in the enzyme activity assays described in Section 2.4.3. Specifically, it includes the manufacturer, catalog numbers, and the detection principles for each kit employed to measure the target enzyme activities. This supporting information is intended to support the reproducibility of the experimental procedures. Please refer to the supporting information document for full details. [file ANU-2026-5561983-s001.docx]

**Supplementary Materials**

**Amylase:**

Manufacturer: Nanjing Jiancheng Bioengineering Institute.

Model: C016-1-1.

Place of origin: Nanjing, China.

Detection methods or principles: Amylase can hydrolyze starch to produce glucose, maltose and dextrin. In the presence of excess known substrate concentration, adding iodine solution forms a blue complex with unhydrolyzed starch. The depth of the blue color can be used to estimate the amount of hydrolyzed starch, thereby calculating the activity of amylase.

**GOT:**

Manufacturer: Nanjing Jiancheng Bioengineering Institute.

Model: C010-2-1.

Place of origin: Nanjing, China.

Detection methods or principles: AST/GOT can convert 2-ketoglutarate and aspartic acid into amino and keto groups, producing glutamic acid and oxaloacetic acid. Oxaloacetic acid can be decarboxylated to pyruvate in the reaction process. Pyruvic acid reacts with 2,4-dinitrophenylhydrazine to produce 2,4-dinitrophenylhydrazone, which shows reddish brown in alkaline solution. After colorimetry, check the standard curve and obtain the activity unit of enzyme.

**GPT:**

Manufacturer: Nanjing Jiancheng Bioengineering Institute.

Model: C009-2-1.

Place of origin: Nanjing, China.

Detection methods or principles: Alanine aminotransferase (ALT) reacts with alanine and α-ketoglutarate to produce pyruvate and glutamic acid at 37℃ and pH 7.4. After 30min of reaction (fixed time), 2,4-dinitrophenylhydrazine (DNPH) hydrochloric acid solution is added to stop the reaction and DNPH adds to carbonyl group of keto acid to produce pyruvate phenylhydrazone. Phenylhydrazone is reddish brown in alkaline condition. The absorbance is read at 505nm and the enzyme activity is calculated.

**Lysozyme:**

Manufacturer: Nanjing Jiancheng Bioengineering Institute.

Model: A050-1-1.

Place of origin: Nanjing, China.

Detection methods or principles: In a certain concentration of turbid bacterial solution, lysozyme can hydrolyze the peptidoglycan on the bacterial cell wall, causing bacterial lysis and the concentration to decrease, leading to an increase in light transmittance. Therefore, the lysozyme activity can be inferred based on changes in light transmittance.

**SOD：**

Manufacturer: Nanjing Jiancheng Bioengineering Institute.

Model: A001-2-2.

Place of origin: Nanjing, China.

Detection methods or principles: Superoxide anion radical (O^2-^·) is produced by xanthine and xanthine oxidase reaction system, and the latter oxidizes hydroxylamine to form nitrite, which shows purple red under the action of chromogenic reagent. Its absorbance is measured by visible spectrophotometer. When the tested sample contains SOD, it has specific inhibition on superoxide anion free radicals, so that the formed nitrite is reduced. The absorbance value of the measuring tube is lower than that of the control tube. The SOD activity in the tested sample can be calculated by formula.

**T-AOC：**

Manufacturer: Nanjing Jiancheng Bioengineering Institute.

Model: A015-2-1.

Place of origin: Nanjing, China.

Detection methods or principles: ABTS is oxidized to green ABTS·+in the presence of suitable oxidants. The production of ABTS·+can be inhibited in the presence of antioxidants. The total antioxidant capacity of the sample can be determined by measuring the absorbance of ABTS·+at 405nm or 734nm. Trolox is an analog of VE, which has similar antioxidant capacity to VE and can be used as a reference for the total antioxidant capacity of other antioxidants. For example, Trolox has a total antioxidant capacity of 1, and the antioxidant capacity of other substances at the same concentration is expressed as a multiple of their antioxidant capacity compared to Trolox.

**Catalase:**

Manufacturer: Nanjing Jiancheng Bioengineering Institute.

Model: A007-1-1.

Place of origin: Nanjing, China.

Detection methods or principles: The reaction of catalase decomposing H_2_O_2_ can be rapidly terminated by adding ammonium molybdate, and the remaining H_2_O_2_ reacts with ammonium molybdate to produce a light yellow complex. The change in absorbance at 405 nm can be measured to calculate the activity of catalase.

**Malondialdehyde (MDA):**

Manufacturer: Nanjing Jiancheng Bioengineering Institute.

Model: A003-1-2.

Place of origin: Nanjing, China.

Detection methods or principles: Malondialdehyde in the degradation products of lipid peroxidation can condense with thiobarbituric acid (TBA) to form a red product with a maximum absorption peak at 532 nm.

**Glutathione peroxidase:**

Manufacturer: Nanjing Jiancheng Bioengineering Institute.

Model: A005-1-2.

Place of origin: Nanjing, China.

Detection methods or principles: Glutathione peroxidase facilitates the reaction between hydrogen peroxide (H_2_O_2_) and reduced glutathione (GSH) to produce water (H_2_O) and oxidized glutathione (GSSG). The activity of glutathione peroxidase can be represented by the rate of its enzymatic reaction. By measuring the consumption of reduced glutathione in this enzymatic reaction, the enzyme activity can be determined.

**[Protein Quantification Kit (BCA Assay)](https://www.abbkine.com/product/protein-quantification-kit-bca-assay-ktd3001/" \o "Protein Quantification Kit (BCA Assay)):**

Manufacturer: Abbkine Scientific Co.,Ltd, China, Wuhan

Model: KTD3001

Detection methods or principles: The principle of this method is that under alkaline conditions, proteins reduce copper ions (Cu2+) to cuprous ions (Cu+). The generated Cu+ forms a purple complex with BCA, which exhibits a strong absorption peak at 562 nm. The absorbance value is directly proportional to the protein content in the sample, and the protein concentration can be calculated based on the absorbance value.

**Protease** (JM-00334F1, Jiangsu Jingmei Biological Technology Co., Ltd, Yancheng, China), **complement 3** (JM-07810F1, Jiangsu Jingmei Biological Technology Co., Ltd, Yancheng, China), **complement 4** (JM-07812F1, Jiangsu Jingmei Biological Technology Co., Ltd, Yancheng, China), **immunoglobulin M** (JM-07517F1, Jiangsu Jingmei Biological Technology Co., Ltd, Yancheng, China), **D-lactic acid** (JM-07610F1, Jiangsu Jingmei Biological Technology Co., Ltd, Yancheng, China), **endothelin-1** (JM-07882F1, Jiangsu Jingmei Biological Technology Co., Ltd, Yancheng, China), **diamine oxidase** (JM-07885F1, Jiangsu Jingmei Biological Technology Co., Ltd, Yancheng, China) were all measured using enzyme-linked immunosorbent assay (ELISA). The principle is based on the specific binding of antigen and antibody. Firstly, specific antigens are coated on the surface of the detection plate. The target substance in the sample (such as proteins) binds to the antigens on the plate. Then, specific antibodies are added to bind to the analyte. Finally, by adding a chromogenic substrate, the concentration of the target substance can be detected based on the color change.

Please refer to the official websites of Nanjing Jiancheng Bioengineering Institute (http://www.njjcbio.com/) and Jiangsu Jingmei Biological Technology Co., Ltd (www.jsjmsw.com) for specific instructions on the reagent kits.
